# A pilot study on the effects of probiotic supplementation on neuropsychological performance and microRNA‐29a‐c levels in antiretroviral‐treated HIV‐1‐infected patients

**DOI:** 10.1002/brb3.756

**Published:** 2017-07-16

**Authors:** Giancarlo Ceccarelli, Mariangela Fratino, Carla Selvaggi, Noemi Giustini, Sara Serafino, Ivan Schietroma, Giuseppe Corano Scheri, Paolo Pavone, Giulia Passavanti, Danilo Alunni Fegatelli, Ivano Mezzaroma, Guido Antonelli, Vincenzo Vullo, Carolina Scagnolari, Gabriella d'Ettorre

**Affiliations:** ^1^ Department of Public Health and Infectious Diseases Sapienza University of Rome Rome Italy; ^2^ Pasteur Institute‐Cenci Bolognetti Foundation Rome Italy; ^3^ Department of Neurology Sapienza University of Rome Italy; ^4^ Department of Molecular Medicine Laboratory of Virology Sapienza University of Rome Italy; ^5^ Department of Clinical Medicine Sapienza University of Rome Italy

**Keywords:** HIV, miRNA, miRNA‐29, neuropsychological performance, probiotics

## Abstract

**Introduction:**

The gut microbiota is involved in the regulation of cognition, mood, anxiety, and pain, and can impact cognitive functions by producing neuroactive substances or releasing bacterial by‐products and metabolites. No information is available on the effects of a probiotic supplementation on brain function of HIV+ subjects. In light of the above considerations, we performed a pilot study in cART‐treated HIV‐1‐positive patients with long‐term virologic suppression. The aims were to analyze the effect of high‐concentration multistrain probiotic supplementation (Vivomixx®; Visbiome®) on several neurocognitive abilities and to evaluate the safety of this supplementation.

**Methods:**

To address those issues, neurocognitive performances were explored by administering neuropsychological tests; moreover, miRNA‐29a‐c levels were measured in cerebrospinal fluid (CSF) to confirm the persistent undetectable levels of HIV‐RNA in the central nervous system after probiotic supplementation.

**Results:**

Our results show that the Rey auditory verbal learning test (RAVLT) (immediate and delayed recall), Rey‐Osterrieth complex figure test (ROCF) (copy immediate and delayed recall), phonological verbal fluency (PVF) test, Toronto alexithymia scale‐20 (Tas‐20), State‐trait anxiety inventory Y‐2 (STAY Y‐2), and time and weight estimation test (STEP) scores improved significantly during the study. Moreover, we found unchanged levels, associated to high degree of individual variability, in miRNA‐29 levels in CSF collected before and after probiotic supplementation.

**Conclusions:**

In conclusion, we observed that HIV patients treated with 6 months of this probiotic supplementation appear to have an improvement in some neurocognitive functions; moreover, this approach is safe and did not modify significantly the levels of miRNA in CSF. Further studies are needed to better understand the contribution of the probiotics in modulating gut–brain‐axis in HIV patients.

## 1 INTRODUCTION

Probiotic supplementation has become a common practice to control the dysbiosis associated to many chronic inflammatory conditions, such as inflammatory bowel diseases (IBD) with positive effects on the clinical outcome (Shen et al., 2014).

HIV is a chronic inflammatory disease in which a proinflammatory change in the gut microflora associated to a number of structural, immunological, and functional changes are present (Ziberman‐Schapira et al., 2016).

Consequently, the administration of probiotics to HIV patients has a rationale, even though the administration of live bacteria in the presence of a viral infection and associated immunosuppression could be a risk for sepsis and abscesses (Haghighat & Crum‐Cianflone, 2015).

Few studies have been published on the role of a probiotic supplementation on SIV+ monkeys and on HIV+ humans, with promising results regarding the immune reconstitution of the gut barrier and immune response (Cunningham‐Rundles et al., 2011; d'Ettorre, Ceccarelli, Andreotti, et al., 2015; d'Ettorre, Ceccarelli, Giustini, et al., 2015; Klatt et al., 2013; Klatt, Funderburg, & Brenchley, 2013; Ortiz et al., 2016; Villar‐García et al., 1999). No information is available on the effects of a probiotic supplementation on brain function and behavior of HIV+ subjects. The gut microbiota is involved in the regulation of cognition, mood, anxiety and pain as previously shown by studies performed in germ‐free animal models or in animals exposed to pathogenic bacterial infections treated by antibiotic drugs or probiotic supplementations (Cryan & Dinan, 2012). The gut microbiota can impact mood and cognitive functions (Desbonnet, Garrett, Clarke, Bienenstock, & Dinan, 2008; Desbonnet et al., 2010) in different ways, i.e., by producing neuroactive substances or releasing bacterial by‐products and metabolites.

In light of the above considerations and the fact that neurocognitive impairment affects more than 50% of HIV‐1‐infected individuals (Nightingale et al., 2014), we performed a pilot study in cART‐treated HIV‐1‐positive patients with long‐term virologic suppression. The aims were (1) to analyze the effect of high‐concentration multistrain probiotic (Vivomixx^®^ Visbiome^®^) supplementation on neuropsychological tests exploring several neurocognitive abilities and (2) to evaluate the safety of this supplementation.

The primary endpoint aimed at evaluating the effect of this specific kind of probiotics supplementation on the following neuropsychological tests: episodic declarative memory (Rey Auditory Verbal Learning Test RAVLT), visual perception and long‐term visual memory function (Rey‐Osterrieth Complex Figure, ROCF), executive function and verbal fluency (Frontal Assessment Battery, FAB; Test of Phonological/Semantic Verbal Fluency, PVF/SVF), working memory's number storage capacity and short‐term memory (Back and Forward Digit‐Verbal Span), visuospatial short‐term working memory (Corsi block‐tapping test, CBTT), visual attention and task switching (Trail making test, TMT), and frontal functions (Time and weight estimation test, STEP).

To evaluate the safety of the product at the neurological level, a lumbar puncture before and after the probiotic supplementation was performed. Due to the invasive procedure of the lumbar puncture and associated risks, a control group was excluded. CSF miRNA‐29a‐c levels were evaluated. miRNAs are short single‐stranded noncoding RNAs that have been shown to play a role in modulating HIV‐1 infection, either directly or indirectly (Monteleone et al., 2015; Müller et al., 2016; Su, Aloi, & Garden, 2016; Swaminathan, Navas‐Martín, & Martín‐García, 2014).

## 2 MATERIALS AND METHODS

### 2.1 Study design, recruitment, and study eligibility criteria

**Figure 1:**
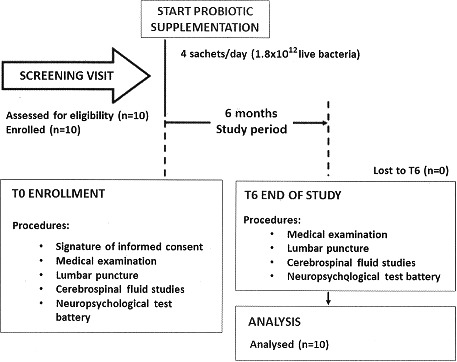
The CONSORT flow diagram of the clinical trial. Experimental design of the study

This is a longitudinal, nonrandomized designed, single‐arm, pilot study (Figure 1) registered on the ClinicalTrials.gov registry with identifier number NCT02276326. The inclusion criteria allowed to enroll HIV‐positive patients (1) who had signed the informed consent, (2) women or men at least 18 years of age, (3) in HAART with HIV‐RNA <50 copies/ml, and CD4 counts >400 cells/mm^3^. Exclusion criteria were: (1) patients with known allergy or intolerance to the product, (2) diarrhea, (3) history of or current inflammatory diseases of the small or large intestine, (4) any past or current systemic malignancy, (5) previous or actual drug addiction, (6) use of antibiotics or probiotics during the 3 weeks prior the enrollment, (7) previous or actual psychiatric disorders, (8) previous or actual neurological disorders, and (9) pregnancy.

Ten HIV‐1‐positive patients successfully treated with ART were recruited at the Division of Infectious Diseases, Department of Public Health and Infectious Diseases, Hospital of “Sapienza” University of Rome (Italy) from May 2014 to February 2015.

All HIV‐1‐infected patients underwent neuropsychological testing and lumbar puncture before (T0) and after (T6) probiotic supplementation of cART, in this way, each patient was the control of oneself. (Figure 1). Because lumbar puncture is considered a procedure with several risks and in literature there is no reported study where lumbar puncture was performed in the control arm, our Institution considered it unethical to have a control group undergoing such a procedure.

Patients received a high‐concentration lyophilized multistrain probiotic supplement twice a day for 6 months (1.8 × 1012 live bacteria/day of a mix of: *Lactobacillus plantarum* DSM 24730, *Streptococcus thermophilus* DSM 24731, *Bifidobacterium breve* DSM 24732, *Lactobacillus paracasei* DSM 24733, *Lactobacillus delbrueckii* subsp. *bulgaricus* DSM 24734, *Lactobacillus acidophilus* DSM 24735, *Bifidobacterium longum* DSM 24736, and *Bifidobacterium infantis* DSM 24737) produced at Danisco Dupont, WI, USA and currently sold in Europe under the brand Vivomixx^®^ and in the USA as Visbiome^®^.

### 2.2 Ethics statement

The researchers declare compliance with ethical practices upon submission of a manuscript and that clinical investigations were conducted according to the principles expressed in the Declaration of Helsinki. The study was approved after amendment (no control group and lumbar puncture to the untreated subjects) by the institutional review board (Department of Public Health and Infectious Diseases, “Sapienza” University of Rome and the Ethics Committee of Umberto I General Hospital, Rome) on 16th January 2014. All study participants gave written informed consent. The authors declare that the Department of Public Health and Infectious Disease where patients were recruited is fully equipped for the planned interventions. The authors confirm that all ongoing and related trials for this drug/intervention are registered.

### 2.3 Cerebrospinal fluid measurements

CSF was collected by lumbar puncture, centrifuged, and cell‐free supernatant samples stored in aliquots at −70°C until analysis of miRNA‐29a/b/c species.

### 2.4 Neuropsychological test battery

All patients underwent an accurate neuropsychological assessment through a complete neuropsychological test battery (Lezak, Howieson, & Loring, 2014). The preliminary cognitive evaluation was based on the administration of Mini‐Mental State Examination (Folstein, Folstein, & McHugh, 1975) (MMSE, score: 0–30) followed by a specific battery of tests to explore the main cognitive domains (memory, attention, executive function, and language) (Belleville, Rouleau, & Caza, 1998; Sannio Fancello, Vio, & Cianchetti, 2006) by means of ROCF Test (Meyers & Meyers, 1995; Rey, 1958) (copy immediate and copy delayed recall, scores, respectively, 0–35 and 0–36), RAVLT (immediate, delayed recall and recognition; scores, respectively: 0–75, 0–15 and 0%–100%), Verbal Span (Digit Forward and Digit Back), FAB (score: 0–18), CBTT (Kessels, van Zandvoort, Postma, Kappelle, & de Haan, 2000) (Forward and Backward), Visual Search Test (Attention Matrices Test) Trail Making Test (Reitan, 1992) (TMTA: mean value 29, insufficient score >78 and TMTB: mean value 75, insufficient score >273), Test of Weights and Measures Estimation (Nichelli et al., 2002) (STEP: Time: score 0–36, normal value 18; Weight: score 0–36, normal value 18; Total: score 0–72, normal value 36), Test of Phonological/Semantic Verbal Fluency (PVF and SVF), Raven's Colored Progressive Matrices (CPM47: nonverbal intelligence), and Aachener Aphasia Test (AAT scores: 0–29 aphasia; >29: normal; <17 serious aphasia). At the same time, to assess psychological manifestations (alexithymia, depression, state and trait anxiety), all patients were evaluated through a set of questionnaires: Toronto Alexithymia Scale‐20 (Bressi et al., 1996) (Tas‐20 score ≤51: non‐alexithymia, ≥61: alexithymia. Scores from 52 to 60: possible alexithymia), Beck Depression Scale II (Storch, Roberti, & Roth, 2004) (BDI‐II, score: 0–63, persistent values around 17 suggest need of treatment), and State‐Trait Anxiety Inventory Y‐1 and Y‐2 (Spinnler & Tognoni, 1987) (STAY Y‐1, Y‐2 scores: 20–80). Where necessary the scores were adjusted for age, sex and instruction levels as required to the normative standards based on the relative Italian validation manuals (Barletta‐Rodolfi, Gasparini, & Ghidoni, 2011). All the tests with the relative validation chosen for our battery, are part of the “category A” according to Bianchi criteria. (Bianchi, 2008). The tests used are standard normalized tests for which normal subject performances are known.

### 2.5 TaqMan‐based real‐time RT‐PCR technique for microRNAs

MicroRNAs were quantified by real‐time RT‐PCR Taqman assays (hsa‐miRNA‐29a, hsa‐miRNA‐29b, hsa‐miRNA‐29c, (Applied Biosystem, Monza, Italy) as previously described (Monteleone et al., 2015). Briefly, miRNAs were extracted from CSF using the Total RNA Purification Kit (Norgen, Thorold, Canada). Then, miRNAs were reverse‐transcripted using a TaqMan MicroRNA Reverse Transcription Kit, according to the manufacturer's protocols (Applied Biosystems). Real‐time PCR was carried out in a final volume of 20 μl using a LightCycler480 instrument (Roche, Basel, Switzerland). The constitutively expressed hsa‐miRNA‐16 and hsa‐mir‐24 were used as internal controls as previously reported. Expression values of miRNA‐29s were calculated by the comparative threshold cycle (CT) method.

### 2.6 Bacterial DNA isolation from fecal samples

Bacterial DNA from fecal samples was extracted using the QIAamp DNA Stool Mini Kit (Qiagen, Hilden, Germany). Approximately 200 mg of feces were cut from frozen samples using sterile disposable scalpel, resuspended in 1.4 ml of ASL lysis buffer from the stool kit, added with glass beads (150–212 μm Sigma‐Aldrich, USA), and homogenized thoroughly. The suspension was incubated at 95°C for 5 min and DNA was purified according to the manufacturer's instructions. DNA was eluted in 200 μl of AE buffer (provided in the kit) and stored at −20°C.

### 2.7 Statistical analysis

Statistical analyses were done using SPSS software, version 20.00 (IBM, Somers, NY, USA). Results obtained from neuropsychological assessment and levels of miRNA‐29 before (T0) and after 6 months of probiotic (T6) supplementation were evaluated using the Wilcoxon test. Differences in MiRNA‐29a‐c expression at T0 and T6 were assessed by the Kruskal–Wallis test. The correlations between MiRNA‐29a‐c levels and neurological test scores were evaluated using the Pearson's test. Differences were considered statistically significant when *p* < .05.

## 3 RESULTS

### 3.1 Demographic and clinical characteristics of HIV‐1‐positive patients

All subjects enrolled were HIV‐1‐infected Caucasian men with a median age of 42 years (22–53 years). They initiated therapy during chronic HIV‐1 infection and had been on cART for a median of 6 years (IQR, 1.75–16.25 years). Pretherapy median CD4+ cell count was 255 cells/mm^3^ (IQR, 42.75–406.75 cells/mm^3^), and zenith HIV‐1 RNA median value was 5.0 log/ml (IQR, 4.81–5.61 log/ml). All subjects had been in virologic suppression (<37 HIV‐1 RNA copies/ml) for at least 1 year and their median CD4+ cell count was 674 (IQR, 564–824 cells/mm^3^) and 683 (IQR, 610–818 cells/mm^3^) cells/mm^3^, respectively, before and after probiotic supplementation of cART. HIV‐RNA and a search for neurotropic viruses were negative in all CSF samples. As reported in the exclusion criteria, no patients enrolled presented previous or actual drug addiction, psychiatric disorders or neurological diseases. Average education level was 12 years.

### 3.2 Neuropsychological performance after probiotic supplementation

A battery of neuropsychological tests was administered to all HIV‐1‐positive patients before (T0) and after 6 months of probiotic supplementation (T6). The tests were readministered after 6 months and a parallel form was used for RAVLT (immediate and delayed recall) the parallel form, according to the Italian validation. (Barletta‐Rodolfi et al., 2011). None of the patients studied had received prior neurocognitive tests.

The results of neuropsychological tests are showed in Table 1. In particular, we observed a significant difference between T0 and T6 for immediate and delayed recall of RAVLT (immediate: *p* = .0273; delayed: *p* = .0299), immediate and delayed recall of ROCF (immediate: *p* = .0058; delayed: *p* = .0019).

**Table 1:** Results of neuropsychological test administered before and after 6 months of probiotic supplementation

	T0 – median (interquartile range)	T6 – median (interquartile range)	*p* value
MMSE (Mini‐Mental State Examination)	30.00 (28.82–30.00)	30.00 (30.00–30.00)	.1814
RAVLT (Rey Auditory Verbal Learning Test Immediate Recall)	46.10 (31.70–47.08)	52.50 (49.35–54.55)	**.0273**
RAVLT (Rey Auditory Verbal Learning Test Delayed Recall)	7.70 (5.92–10.30)	12.00 (10.38–12.48)	**.0299**
RAVLT (Rey Auditory Verbal Learning Recognition)	95.50 (90.00–99.50)	99.50 (98.00–100.00)	.1056
ROCF (Rey‐Osterrieth Complex Figure Copy Immediate recall)	16.30 (15.92–17.03)	21.00 (18.58–22.95)	**.0058**
ROCF (Rey‐Osterrieth Complex Figure Copy Delayed Recall)	15.25 (14.32–16.75)	22.40 (22.00–24.75)	**.0019**
FAB (Frontal Assessment Battery)	14.85 (13.60–15.80)	15.45 (14.21–17.50)	.1780
PVF (Phonological Verbal Fluency)	28.50 (22.82–37.88)	43.95 (42.25–44.75)	**.0137**
SVF (Semantic Verbal Fluency)	47.00 (32.75–54.75)	48.50 (45.50–49.00)	.3580
TMTA (Trail Making Test A)	50.00 (44.25–55.00)	46.00 (40.50–54.00)	.1395
TMTB (Trail Making Test B)	117.50 (99.50–122.80)	125.00 (98.50–138.50)	.9527
Verbal Span‐Digit Forward	4.50 (3.50–5.37)	5.00 (5.00–5.81)	.0940
Verbal Span‐Digit Back	4.50 (4.00–5.00)	5.00 (4.25–5.00)	.5716
Tas‐20 (Toronto Alexithymia Scale‐20)	40.00 (35.00–41.00)	50.00 (41.00–54.00)	**.0421**
BDI‐II (Beck Depression Scale II)			
STAY Y‐1 (State‐Trait Anxiety Inventory)	47.00 (45.00–50.00)	55.00 (50.00–70.00)	.0751
STAY Y‐2 (State‐Trait Anxiety Inventory)	60.00 (40.00–65.00)	85.00 (65.00–90.00)	**.0225**
AAT (Aachen Aphasia Test)	9.00 (9.00–9.00)	9.00 (9.00–9.00)	1.0000
CBTT‐ Forward (Corsi block‐tapping task)	4.37 (4.00–5.00)	5.25 (5.05–5.44)	**.0322**
CBTT‐Backward (Corsi block‐tapping task)	4.00 (3.00–4.00)	4.00 (4.00–4.75)	.1198
STEP Time (Time and Weight Estimation test)	16.50 (13.25–22.25)	23.00 (21.25–23.00)	**.0246**
STEP Weight (Time and Weight Estimation test)	19.00 (15.00–19.75)	21.00 (21.00–23.00)	**.0220**
STEP Total (Time and Weight Estimation test)	36.50 (29.75–44.25)	44.50 (43.00–45.00)	.0827

Significantly different values are highlighted in bold.

Also, PVF (*p* = .0137), STEP (time: *p* = .0246; weight: *p* = .022), Tas‐20 (*p* = .0421), STAY Y‐2 (*p* = .0225), and CBTT‐Forward (*p* = .0322) test scores increased significantly in HIV‐1‐positive patients who took probiotics twice a day for 6 months.

By contrast, no significant differences were observed for the other neuropsychological test scores examined before and after probiotic intake.

### 3.3 Expression of microRNA‐29a‐c

The analysis of miRNA‐29a‐c levels in CSF collected from HIV‐1‐infected patients before (T0) and after 6 months of daily (T6) probiotic supplementation showed a high degree of individual variability in miRNA‐29 levels [coefficient of variation (%): T0 (miRNA‐29a: 91.30; miRNA‐29b: 135.858; miRNA‐29c: 119.93); T6 (miRNA‐29a: 118.54; miRNA‐29b: 156.46; miRNA‐29c: 160.32)]. After probiotic supplementation, miRNA‐29a‐c levels and the pattern of miRNA‐29 expression remained unchanged.

### 3.4 Bacterial DNA isolation from fecal samples

We found that fecal Bifidobacteria spp. increased significantly in all patients compared to their basal level, after 2 months of supplementation (*p* < .05) and remained stable through the study (data not shown) and this was considered as a proof of compliance to the treatment.

### 3.5 Safety of probiotic supplementation

All patients received a high‐concentration lyophilized multistrain probiotic powder supplement twice a day for 6 months (1.8 × 10^9^). This dosage was well‐tolerated throughout the course of the study and side effects were not reported.

## 4 DISCUSSION

Neurocognitive disorder affects more than 50% of antiretroviral‐treated HIV subjects (Heaton et al., 2010). An improvement, but not complete recovery, of neurocognitive impairment has been reported in the literature, but only after the cART was intensified which often is not a feasible avenue due to the untoward effects and chronic nature of the disease (Stilling, Dinan, & Cryan, 2014). We hypothesized that a new and safe approach for the improvement of the neuropsychological performance of these subjects could be the administration of probiotics. In the last years, there has been an increase in experimental research, conducted mainly on animals, aimed to explore the contribution of the microbiota in modulating gut–brain‐axis (GBA). The discovery that differential microbial composition is associated with alterations in behavior and cognition has significantly contributed to establish the “microbiota–gut–brain‐axis” as an extension of the well‐accepted “gut–brain‐axis” concept. This concept is used to describe the bidirectional communication between the CNS and intestinal organs (Gates et al., 2016).

Recent studies exploring the effects of different gut microbiota composition on impairment of cognition and behavior have shown that alterations of microbioma composition are connected to stress, modification of mood and behavior, worsening of cognition and that probiotics can play a protective role (Carabotti, Scirocco, Maselli, & Severi, 2015; Jiang et al., 2015; Steenbergen, Sellaro, van Hemert, Bosch, & Colzato, 2015).

Since no data were available, our primary study end point was to verify the safety of a high‐concentration multistrain probiotic supplementation at the neurological level. Each patient underwent a lumbar puncture before and after probiotic supplementation to verify any changes in CSF.

All HIV‐1‐positive patients received a high‐concentration lyophilized multistrain probiotic powder supplement twice a day for 6 months (1.8 × 10^9). This dosage was well‐tolerated throughout the course of the study and the potential side effects which had been previously reported for other and different formulations were not reported here (Anukam, Osazuwa, Osadolor, Bruce, & Reid, 2008; Haghighat & Crum‐Cianflone, 2015; Hummelen et al., 2011). We found that fecal *Bifidobacteria* spp increased in all patients compared to their basal level, at 2 months of supplementation and remained stable throughout the study confirming adherence of the patients to the regimen.

Our results showed that probiotics do not affect miRNA‐29a‐c levels or the signature of miRNA‐29‐a‐c in CSF collected from cART‐fully suppressed HIV‐1‐positive patients confirming the neurological safety of the probiotic formulation, a finding never described before in the literature.

Moreover, our patients presented a relevant improvement of performance in the neuropsychological and behavioral tests after 6 months of probiotic intake. MMSE was not modified by 6 months of probiotic supplementation while RAVLT (immediate and delayed recall), ROCF (immediate and delayed recall), PVF, STAY Y‐2, and STEP (total, time and weight) appeared significantly improved.

Our data seem to demonstrate that the cognitive domains more involved are the short and long memory and the abstract reasoning (one of the aspects involved in the executive functions). Such results, seem to be aligned with what has emerged from a previously study, in which it has been highlighted that a crucial role in the bidirectional communication of the gut–brain‐axis, is covered by the hypothalamic–pituitary adrenal (HPA) axis. As well known, the HPA axis is considered the core stress efferent axis that coordinates the adaptive responses of the organism to stressors of any kind, and also it is a part of the limbic system, a crucial zone of the brain predominantly involved in memory and emotional responses (Carabotti et al., 2015; Gates et al., 2016).

Pending the confirmation by larger study, probiotics supplementation seems to be a safe dietary approach to improve neurocognitive performance in HIV‐1‐positive patients, in particular memory functions. The same multistrain high‐concentration probiotic has been shown to increase the brain‐derived neurotrophic factor (BDNF) expression and to attenuate age‐related alterations in the hippocampus of rats (Reitan, 1992).

On a pure speculative basis, according to what has been observed in animal models, probiotics may act on the hippocampal regions controlling memory functions in HIV‐positive subjects undergoing antiretroviral treatment (Mayer et al., 2015), so the manipulation of microbial taxa trough probiotics may provide a means by which cognition may be altered in a reproducible and consistent manner in order to achieve a beneficial outcome for the patients in all daily activities in which, are required cognitive functions as memory (Lyte et al., 2016).

Significant variations have been seen on the TAS‐20 that evaluate the level of alexithymia and on the STAY‐Y2 test, that on the other hand, in used to measure the level of the anxiety of the patients. In our opinion, based on clinical observation and clinical interviews, the increase of these variables could be due to an increased awareness of the underlying disease, also reported by the patients themselves, leading also to the improvement of cognitive functions. However, this hypothesis needs further observations.

On the basis of our results, HIV‐positive patients treated with probiotic supplementation improved their performance significantly in some neurocognitive domains (short and long memory and abstract reasoning), while their abilities did not vary in the other domains explored. At this moment, the origin of this clinical observation is unknown. As previous reported, possible reason might be due to the fact that hippocampal regions are involved only in certain aspects of neurocognitive processes, but further studies are needed to understand this topic. Moreover, we cannot rule out that the power of statistical analysis has been reduced by the sample size of the cohort studied.

This study suffers for the small sample size and the related statistical concerns and lacks proper control for multiple correlations. However, the small sample size is justified by the need to verify the safety of this high‐concentration multistrain probiotic supplementation. Usually, few thousand million probiotic bacteria have been administered to AIDS patients and a prudent approach (pilot study) consequently was mandatory. It is evident that a small sample size limits the capability to analyze properly the results from a statistical point of view. However, in the field of HIV many studies have been published even when the number of patients was limited and no control group was enrolled as their observations and results prompted larger confirmatory trials. Moreover, another limitation of our study was the absence of a control group. The reasons that explain the absence of a control group are essentially related to restrictions indicated by the ethics committee, namely: (1) lumbar puncture presents several risks (i.e., seeding of infection to the CSF; epidermoid tumor implantation; uncal or transtentorial herniation and neurological deterioration; spinal hematoma), for these reasons, performing this procedure on a control group of healthy subjects was not considered acceptable by the ethics committee; (2) in addition, the use of placebo in HIV patients under heavy cART medication is discouraged;, and (3) last but not least, since the product we administered is a probiotic, the placebo group should have been on a dietary restriction for all the fermented foods containing bacterial species (yogurt, kefir, sour cream, cheese, crème fraiche, salami, vinegar, fish sauce, etc.). Unnecessary dietary restrictions to HIV‐positive subjects are not considered ethically acceptable by our Institution.

Furthermore, although our data suggest the improvement of some cognitive domains, we cannot totally exclude the possible influence of the practical effect on neuropsychological performances. The use of parallel/alternate forms is one possible approach to minimize practice effect, but it does not fully eliminate concerns about this topic. Our findings seem to be encouraging, but future studies, carried out with specific methodological procedures, are needed to support their validity.

In conclusion, despite the above limitations justified by the complexity of the procedures and the ethical boundaries, the findings of this pilot study indicate that HIV‐1‐positive patients receiving probiotic formulation twice a day for 6 months had significantly improved some neurological cognitive functions (short and long memory and abstract reasoning), thus supporting the concept that modifications of the gut flora can provide specific neurological benefits in HIV‐1 patients in the absence of significant side effects.

## CONFLICTS OF INTEREST

The authors declare no conflict of interest.
